# Salivary IgA and dentine caries in relation to physical activity and fitness: A cohort-based cross-sectional study

**DOI:** 10.1007/s00784-026-07035-y

**Published:** 2026-07-15

**Authors:** Eero Blomster, Marja-Liisa Laitala, Sohvi Hörkkö, Ramin Akhi, Tarja Tanner

**Affiliations:** 1 Department of Cariology, Endodontology, and Paediatric Dentistry, Research Unit of Population Health, University of Oulu, Oulu, Finland; 2 Medical Research Center, University of Oulu and Oulu University Hospital, Oulu, Finland; 3 Medical Microbiology and Immunology, Research Unit of Biomedicine and Internal Medicine, University of Oulu, Oulu, Finland

**Keywords:** Dental caries, Saliva, Immunoglobulins, Physical activity, Physical fitness, Oral health

## Abstract

**Objectives:**

Emerging evidence suggests that higher physical activity and physical fitness are associated with fewer decayed teeth (DT). This study investigates whether salivary immunoglobulin A (SIgA) mediates the association between physical activity or fitness and DT, and whether dentine caries is associated with salivary and systemic immunoglobulin levels in adults.

**Materials and methods:**

Data were derived from the 46-year follow-up of the Northern Finland Birth Cohort 1966. Participants (*N* = 1,589) underwent clinical oral examinations using ICDAS criteria. SIgA and serum immunoglobulins (IgA, IgG, IgM) were analysed from saliva and fasting blood samples using a chemiluminescence immunoassay. Physical activity was measured objectively using wrist-worn accelerometers, and physical fitness by step testing and heart rate recovery. Mediation analyses examined whether SIgA mediated associations between physical activity or fitness and DT. Associations between immunoglobulins and DT were analysed using adjusted negative binomial regression.

**Results:**

Descriptive analyses indicated higher SIgA levels among participants with greater dentine caries burden. No significant indirect effects of physical activity or fitness on caries through SIgA were observed. In adjusted models, higher SIgA concentrations were associated with increased DT (Exp(β) = 1.282, *p* = 0.001), whereas serum IgA, IgG, and IgM were not associated with caries.

**Conclusions:**

No evidence was found that SIgA statistically mediated the association between physical activity or fitness and dentine caries in this dataset. Higher SIgA levels are associated with greater caries burden, suggesting a reactive rather than protective role.

**Clinical relevance:**

Salivary SIgA may reflect immune activation in response to dentine caries rather than protection against disease, highlighting the potential role of local immunity in caries progression.

**Supplementary Information:**

The online version contains supplementary material available at 10.1007/s00784-026-07035-y.

## Introduction

Dental caries and physical inactivity are prevalent global health problems that substantially contribute to the burden of non-communicable diseases and rising health and economic costs worldwide [1, 2]. Despite this, the potential biological links between these conditions remain insufficiently defined.

In the previous cohort study among middle-aged Finnish population, high levels of physical activity and better physical fitness were associated with a lower prevalence of dentine caries [3]. Similar findings have also been reported elsewhere [4]. This raises the question of whether the association is mediated solely by behavioral factors such as oral health habits and diet, which commonly accompany more active lifestyles [5], or whether biological mechanisms may also contribute.

Salivary immunoglobulin A (SIgA), the main immunoglobulin in saliva, is a key component of mucosal immunity, preventing microbial adhesion and neutralizing toxins [6]. Low SIgA levels have been associated with increased infection risk, particularly among individuals undergoing intense physical training [7, 8]. While SIgA is crucial to oral immunity, serum immunoglobulins such as IgA, IgG, and IgM contribute to systemic immune responses through pathogen neutralization and inflammation regulation [9–11].

Evidence on the association between SIgA concentration and dental caries is sparse and inconsistent. While some studies with small samples suggest a compensatory increase in SIgA with a higher caries burden [12, 13], most support a protective role with lower levels in caries-active individuals [14–16]. A recent meta-analysis supports the latter, indicating lower SIgA levels in those with more caries [17]. Unlike SIgA, IgG and IgM enter the oral cavity mainly via gingival crevicular fluid and are present in lower concentrations, making their contribution to salivary immune defense uncertain [18]. The role of systemic antibodies in dental caries remains largely unexplored, unlike in periodontitis, where immune responses are better characterized [19].

Physical activity and fitness have well-established modulatory effects on immune function. Moderate exercise enhances SIgA secretion, while prolonged or intensive activity can temporarily suppress mucosal immunity [20]. Physical activity also activates the autonomic nervous system, which may alter salivary gland function and antibody secretion [21]. Because SIgA plays a role in mucosal immunity by limiting microbial adhesion, exercise-related changes in SIgA secretion could influence susceptibility to dental caries. Therefore, SIgA could represent a potential biological pathway linking physical activity or fitness with dental caries. The effects on systemic immunoglobulin levels are less consistent. Some studies report transient reductions in serum IgA, IgG, and IgM after intense exercise [22], while others find no significant changes [23].

Despite these insights, limited evidence exists on whether SIgA mediates the association between physical activity, physical fitness, and dentine caries, particularly in adults. Furthermore, little is known about the associations between dentine caries and systemic humoral immune responses.

Therefore, the primary aim of this study was to evaluate whether SIgA statistically mediates the association between physical activity or physical fitness and dentine caries in this cohort. The secondary aim was to assess the associations of salivary and systemic immunoglobulins with DT in 46-year-old adults from the Northern Finland Birth Cohort 1966.

## Methods

### Study population

The Northern Finland Birth Cohort 1966 [24] was established to follow individuals born in the two northernmost provinces of Finland, Oulu and Lapland, with an expected birthdate in 1966 (*N* = 12,231). The cohort has been monitored through repeated clinical examinations and health-related questionnaires at various ages [24].

For the 46-year follow-up, conducted between 2012 and 2014, all cohort members (*N* = 10,331) were invited to participate in a comprehensive health examination carried out in 36 locations across Finland. Among them, a subgroup of 3,181 participants residing within 100 km of Oulu was invited for a clinical oral examination [25]. A total of 1,962 individuals (62.3%) participated in this examination.

**Fig. 1:**
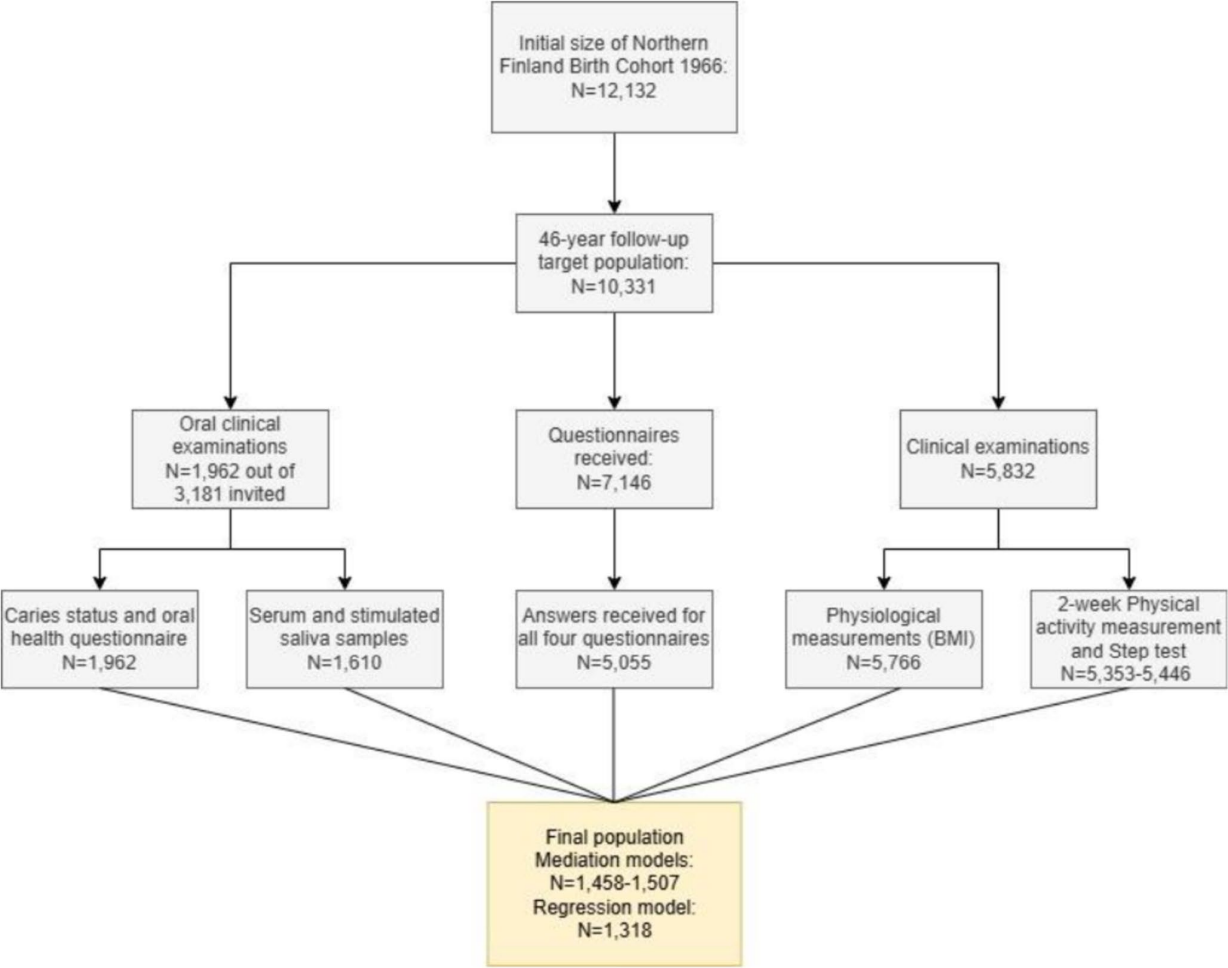
Flowchart of data collection and participant inclusion for the 46-year follow-up of the Northern Finland Birth Cohort 1966

This study analyzed data from participants in the 46-year follow-up of the Northern Finland Birth Cohort 1966 who had complete data available, including saliva and serum samples, oral clinical examination results, and relevant questionnaire and clinical health information. The full flow of participants is presented in Fig. 1.

Descriptive analyses (Table 1; Fig. 3) were conducted among the 1,589 participants who provided stimulated saliva samples.

**Table 1:** Characteristics of participants with and without available salivary samples in the NFBC1966 46-year follow-up

Categorical variables	Category		Saliva samples available *n* = 1,*589*		Saliva samples unavailable *n* = 5,*515*	
*Gender*	Male		736 (46.5%)		2,547 (46.2%)	
	Female		847 (53.5%)		2,968 (53.8%)	
	Total		1,583 (99.6%)		5,515 (100%)	
*BMI*	< 18.5		7 (0.4%)		29 (0.7%)	
	18.5–24.9		627 (39.6%)		1,594 (38.2%)	
	25-29.9		621 (39.2%)		1,648 (39.4%)	
	≥ 30		327 (20.7%)		907 (21.7%)	
	Total		1,582 (99.6%)		4,178 (75.8%)	
*Smoking*	No		1,216 (79.3%)		3,771 (73.0%)	
	Yes		318 (20.7%)		1,396 (27.0%)	
	Total		1,534 (96.5%)		5,515 (100%)	
*Daily tooth* *brushing frequency*	≤ 1		440 (29.4%)		105 (30.7%)	
	2		1,013 (67.6%)		226 (66.1%)	
	≥ 3		45 (3.0%)		11 (3.2%)	
	Total		1,498 (94.3%)		342 (6.2%)	
*Dentine caries lesions (ICDAS 4–6)*	0		947 (60.8%)		207 (57.3%)	
	> 0		610 (39.2%)		154 (42.7%)	
	Total		1,557 (98.0%)		361 (6.5%)	
*Snacks consumption* ^*a*^	Low		741 (48.6%)		180 (50.4%)	
	Moderate		689 (45.2%)		149 (41.7%)	
	High		95 (6.2%)		28 (7.8%)	
	Total		1,525 (96.0%)		357 (6.5%)	
*Continuous variables*	*Saliva samples available* *n* = 1,*589*			*Saliva samples unavailable* *n* = 5,*515*		
	*Mean (SD)*	*Total*		*Mean (SD)*		*Total*
*DMFT score*	14.82 (5.11)	1,557		15.20 (5.38)		362
*Dentine caries lesions (ICDAS 4–6)*	0.93 (1.79)	1,557		1.21 (2.07)		361
*Enamel caries surfaces (ICDAS 1–3)*	29.34 (14.99)	1,558		30.18 (15.60)		361
*Physical fitness* ^*b*^	40.47 (10.80)	1,479		41.04 (11.48)		3,713
*Physical activity* ^*c*^	31.63 (20.55)	1,529		31.71 (20.84)		3,912
*Annual household income (€)* ^*d*^	68,537 (70,613.38)	1,416		70,715 (302,814.71)		4,597

^a^Self-reported consumption of beverages, candy, or snacks. Higher value indicates more frequent consumption

^b^Measured as heart rate recovery in bpm in the 60 s after a step test normalized to peak heart rate

^c^Amount of vigorous and very vigorous activity, measured by MET-values and reported as minutes/day

^d^Total household income before taxes

Percentages for category rows are calculated among participants with available data for that variable. Total rows indicate the number and proportion of participants with available data

The variation in sample size reflects differences in the covariates used across analyses. Missing data were not imputed. The study was approved by the Northern Ostrobothnia Hospital District Ethical Committee (94/2011, 12.12.2011), and all participants provided written informed consent.

### Oral health examination, questionnaires, and oral health-related variables

The clinical oral examinations were conducted at the Institute of Dentistry, Faculty of Medicine, University of Oulu, Finland, in a modern dental clinic setting. Seven calibrated dentists, who had undergone specialized training in the study protocols, performed the examinations following a standardized protocol. A dental nurse recorded the findings in an electronic patient file. To ensure consistency, a senior researcher served as the gold standard for validation [24].

The International Caries Detection and Assessment System (ICDAS) [26] was applied to assess caries at the tooth surface level. Tooth surfaces with ICDAS scores of 4–6 were classified as dentine caries lesions requiring restorative treatment. A tooth was classified as having dentine caries if at least one of its surfaces received an ICDAS score of 4–6. DT was then calculated as the total number of teeth with at least one ICDAS 4–6 lesion. Consistent with our previous NFBC1966 study [3], dentine caries (ICDAS 4–6) was used as the primary outcome variable in the present analyses. All participants in the dental clinical examination also completed a computer-administered oral health questionnaire [25], from which data on tooth brushing frequency were used in this study. Additionally, data were obtained from separate postal questionnaires on tobacco use, based on the following three questions: “Have you ever smoked during your lifetime?”, “Do you currently smoke”, and “When was the last time you smoked?”, as well as total household income and dietary habits, based on the question: “Do you think you usually consume soft drinks, sweets, or other snack treats?”.

### Immunoglobulins level measurement

Salivary IgA (SIgA) was measured from stimulated saliva samples. Participants were instructed to chew a paraffin block for five minutes, during which saliva was collected. Samples were centrifuged at 1200 rpm for 20 min at 4 °C, after which supernatant and pellet were separated and stored at − 80 °C until analysis [27, 28].

Participants attended the study visit according to a standardized and scheduled examination protocol, during which measurements were performed in a fixed order. Saliva sampling was conducted under controlled conditions within this protocol, with consideration given to recent food intake, smoking, and physical activity to minimize acute influences on salivary biomarkers.

Systemic immunoglobulins were collected from the antecubital vein after an overnight fast, with participants in a supine position. All salivary and serum immunoglobulin levels (IgA, IgG, IgM) were determined using a chemiluminescence immunoassay. To ensure analytical reliability, all samples were measured in duplicate, and standard curves of purified human immunoglobulins were used to control for inter-assay variability.

Stimulated saliva was used due to sample availability in the cohort. SIgA data were available from 1,589 participants and serum immunoglobulins from 1,591 of 1,962 participants. Detailed sampling procedures and laboratory analyses have been described previously [27, 29].

### Physical fitness and physical activity variables

Physical fitness was assessed using a step test in which participants stepped on a 30 cm platform at a metronome-paced rhythm for three minutes and were evaluated based on heart rate recovery (HRR) measured 60 s post-test. Physical activity was measured with an accelerometer worn on the non-dominant hand for 14 days, excluding water activities [30]. The accelerometer assessed metabolic equivalent of task (MET) values [31] every 30 s using height, weight, gender, and age as predefined inputs, and categorized physical activity into five intensity levels (Very light to Very vigorous). Valid physical activity data required ≥ 4 days with at least 600 min/day of wear time.

### Statistical analysis

**Fig. 2:**
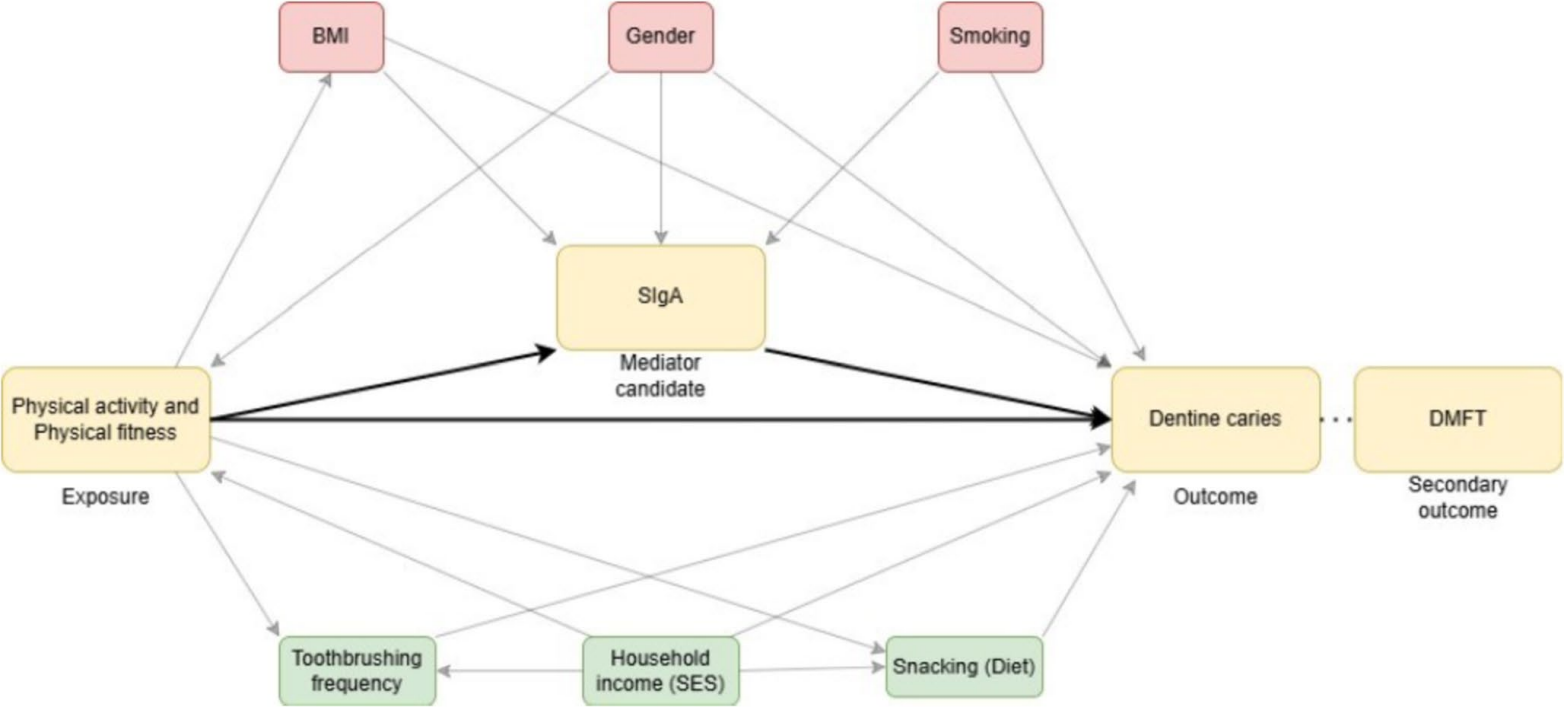
Directed acyclic graph (DAG) of the association between physical activity/fitness, SIgA, and dentine caries. SIgA is modeled as a potential mediator, with BMI, gender, smoking, oral hygiene, socioeconomic status, and diet as confounders; DMFT is included as a secondary outcome

A directed acyclic graph (DAG) was constructed to illustrate the hypothesized relationships between physical activity and fitness, SIgA, dentine caries, and covariates, and to support covariate selection for the adjusted analyses (Fig. 2).

**Fig. 3:**
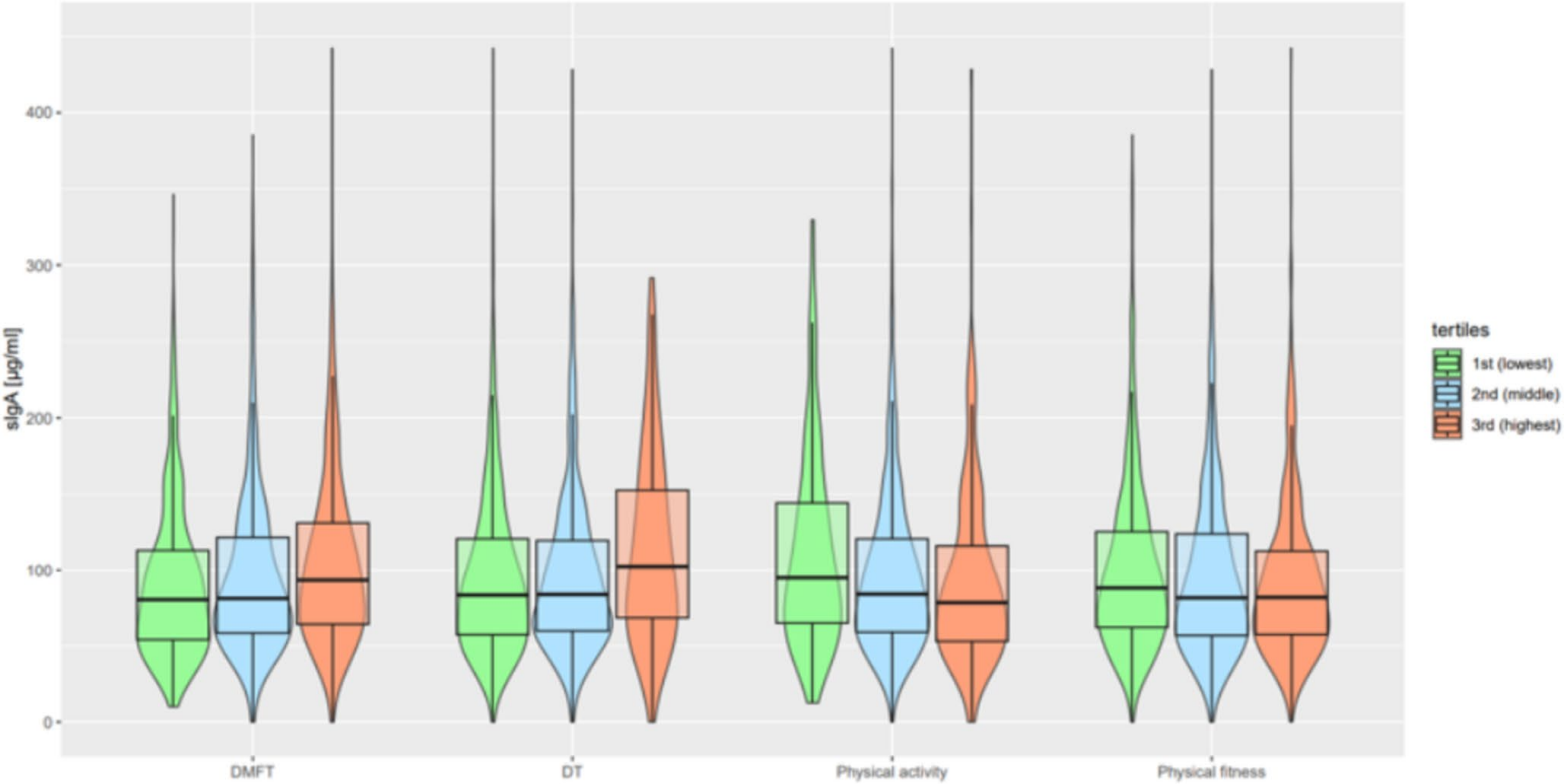
Distribution of salivary IgA levels by three categories of DMFT, Tooth decay (ICDAS > 3), Physical activity, and Physical fitness. DMFT (Decayed, Missing, and Filled Teeth) was categorized to DMFT < 11 (*N* = 326), 11–19 (*N* = 929), and > 19 (*N* = 302), and DT (Decayed teeth) to 0 (*N* = 947), 1–3 (*N* = 493), and > 3 (*N* = 117) dentine caries lesions. Physical activity and physical fitness were categorized as the lowest 10%, middle 80%, and highest 10% of the distribution. Total sample sizes varied between analyses (*N* = 1,479–1,557)

SIgA concentrations were explored descriptively across categorized levels of DT, physical activity, and physical fitness using boxplots (Fig. 3). Dentine caries history was assessed using the DMFT index and categorized into three groups: values under 11 (lowest), 11 to 19 (middle), and over 19 (highest), and was considered a secondary outcome. DT was categorized as follows: 0 (lowest), 1 to 3 (middle), and over 3 (highest). Physical fitness was categorized into three groups using a 10-80-10% distribution: 0–27 bpm (lowest), 28–53 bpm (middle), and 54–87 bpm (highest). Physical activity was based on the total daily duration (minutes) of vigorous and very vigorous activity (≥ 6 MET), and similarly divided into the lowest 10%, middle 80%, and highest 10%.

**Fig. 4:**
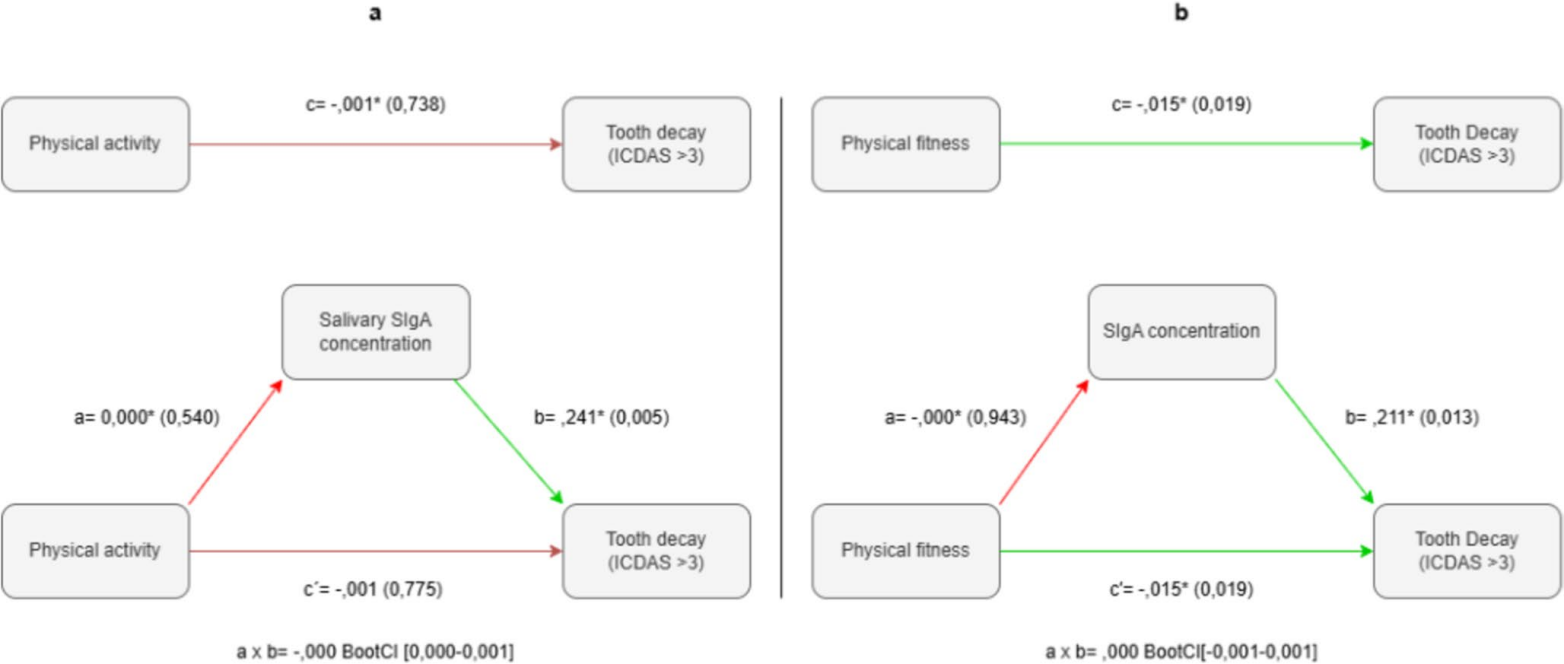
Bootstrapped mediation models testing the indirect effects of physical activity (**a**) and physical fitness (**b**) on dentine caries (ICDAS > 3) through salivary IgA concentration from stimulated saliva, adjusted for gender, BMI, and additional covariates related to lifestyle and oral health behavior (tooth brushing frequency, tobacco use, snacking, and household income). Path a represents the association between the exposure (physical activity/fitness) and the mediator (SIgA), path b the association between SIgA and dentine caries, and c the total effect of the exposure on dentine caries. Path c′ represents the direct effect after accounting for the mediator. The indirect effect is calculated as a × b. Standardized regression coefficients (β) are shown along each path, with p-values in parentheses, and bootstrapped confidence intervals (BootCI) for the indirect effect

To examine whether SIgA mediates the association between physical activity or fitness and DT, bootstrap-based mediation analyses were performed (Fig. 4). These analyses assessed the indirect effect of physical activity and fitness on DT through SIgA, using 5,000 bootstrap samples to estimate confidence intervals for the mediated effect. Gender, BMI, Tobacco usage, Snacking frequency, Tooth brushing frequency, and household income were included as covariates in the models.

To investigate whether DT was associated with salivary and systemic antibody responses (Table 2), a negative binomial regression model was conducted with log-transformed levels of SIgA, serum IgA, IgG, and IgM as predictors of DT (continuous variables), while controlling for potential confounders including body mass index (BMI), tobacco use, snacking frequency, total household income, tooth brushing frequency, and gender. BMI was calculated as weight in kilograms divided by height in meters squared (kg/m²) and analyzed as a continuous variable. Tobacco use was categorized as No (non-smoker or former smoker who quit ≥ 6 months ago) and Yes (former smoker who quit 1–6 months ago or current smoker). Socioeconomic status (SES), also analyzed as a continuous variable, was based on total annual household income before taxes. Snacking frequency was categorized as Less than others/ The same as others/ and More than others. Tooth brushing frequency and snacking frequency were treated as ordinal variables in the regression models to assess linear trends across categories.

**Table 2:** Negative binomial regression model of Tooth decay (DT) (ICDAS > 3) to compare the associations of dentine caries and the concentrations of salivary and systemic antibodies (*N* = 1,318)

Predictor variables of DT	Exp(β)	*p*-value	95% CI
*Salivary IgA (SIgA)* ^*a*^	1.282	0.001	(1.103, 1.490)
*Serum IgA*	0.809	0.082	(0.638, 1.027)
*Serum IgG*	1.218	0.141	(0.936, 1.584)
*Serum IgM*	1.122	0.150	(0.959, 1.313)
*Tooth brushing frequency*	0.663	< 0.001	(0.563, 0.780)
*Tobacco usage*	1.157	< 0.001	(1.079, 1.241)
*Household income*	0.850	< 0.001	(0.785, 0.921)
*Gender* ^*b*^	0.744	0.001	(0.623, 0.889)
*BMI*	0.992	0.343	(0.974, 1.009)
*Snacks* ^*c*^	1.043	0.546	(0.910, 1.194)

^a^Salivary IgA concentration from stimulated saliva samples

^b^For Gender: 1 = Male and 2 = Female

^c^Self-reported consumption of beverages, candy, or snacks. Higher value indicates more frequent consumption

SIgA was log-transformed in all adjusted models (Fig. 4; Table 2) to improve distributional properties and reduce skewness, while DT, physical activity, and physical fitness were treated as continuous variables. Negative binomial regression was selected because DT was a count variable exhibiting overdispersion, making it more appropriate than a Poisson model.

Results from the mediation analyses were presented as standardized regression coefficients (β) and p-values, with statistical significance defined as *p* < 0.05, whereas results from the negative binomial regression model were presented as exponentiated coefficients (Exp(β)) with 95% confidence intervals. Bootstrapped confidence intervals (BootCI) were reported for the mediation analyses. Statistical analyses were performed using IBM SPSS Statistics for Windows (version 29.0.0.0, Armonk, NY: IBM Corp.). Mediation analyses were conducted using the PROCESS macro (version 4.2) by Andrew F. Hayes for SPSS. Boxplots were generated using RStudio (version 2024.12.0 + 467, 2024-12-11) for Windows.

This study adheres to the STROBE guidelines.

## Results

### Cohort profile

Among 1,589 participants in this birth cohort study, 53.5% were female and 59.9% had a BMI ≥ 25 kg/m². Current or occasional smoking was reported by 20.7%. The mean pre-tax annual household income was €68,537. Only two participants had DMFT = 0 and the mean DMFT was 14.82. By contrast, 60.8% had DT = 0, and the mean DT was 0.93 (Table 1). Taken together, these data indicate that, at midlife, this population shows substantial cumulative caries experience but relatively low current untreated dentine lesions.

### SIgA is positively associated with caries burden and inversely associated with physical activity

Next, we explored whether salivary IgA (SIgA) varied across levels of caries, physical activity, and fitness. Figure 3 illustrates the distribution of SIgA concentrations across categories of current dentine caries (DT), cumulative caries experience (DMFT), physical activity, and physical fitness. Visually, higher SIgA levels tended to occur among individuals with greater caries burden, whereas slightly lower levels were observed among participants with higher physical activity. Differences across physical fitness categories appeared small. The corresponding ANOVA and post-hoc comparisons are presented in Supplementary Table S1. These descriptive patterns motivated further evaluation using regression and mediation models.

### No evidence of salivary IgA mediation in the physical activity–caries association

In the first mediation model (Fig. 4a), the total effect of physical activity on DT was not significant (c = –0.001, *p* = 0.738), nor was the direct effect after accounting for SIgA (c′ = –0.001, *p* = 0.775). Although SIgA concentration was significantly associated with DT (b = 0.241, *p* = 0.005), physical activity was not significantly associated with SIgA levels (a = 0.000, *p* = 0.540), and the indirect effect via SIgA was not statistically significant (a×b = 0.000; 95% BootCI [0.000, 0.001]), providing no evidence of mediation.

In the second mediation model (Fig. 4b), physical fitness showed a significant total effect on DT (c = –0.015, *p* = 0.019), as well as a significant direct effect after accounting for SIgA (c′ = –0.015, *p* = 0.019). SIgA was again significantly associated with DT (b = 0.211, *p* = 0.013), but the association between physical fitness and SIgA was not significant (a = –0.000, *p* = 0.943). The indirect effect remained nonsignificant (a×b = 0.000; 95% BootCI [–0.001, 0.001]), again providing no evidence of mediation.

### Salivary IgA is associated with decayed teeth in the adjusted model

In the negative binomial regression model, only SIgA concentration emerged as a significant predictor of DT (Exp(β) = 1.282, *p* = 0.001). In contrast, serum IgA (*p* = 0.082), IgG (*p* = 0.141), and IgM (*p* = 0.150) were not significantly associated with DT. Among the covariates, lower tooth brushing frequency, tobacco use, lower household income, and male gender were also significantly associated with a higher number of decayed teeth (Table 2).

## Discussion

In this middle-age birth cohort, descriptive analyses indicated higher SIgA levels among participants with greater dentine caries burden (DT) and cumulative caries experience (DMFT). In the mediation models, DT was positively associated with SIgA, while neither physical activity nor physical fitness showed significant associations with SIgA. In our previous work from the current cohort, we established that both higher physical activity and fitness were associated with a more than 1.5-fold reduction in DT [3]. Here, multivariable models identified SIgA as the only immunologic variable independently predicting DT, as serum IgA, IgG, and IgM showed no association with current caries. Together, these findings suggest that mucosal immune responses may be more closely associated with active dentine lesions than systemic immunoglobulin levels in this dataset.

In the Introduction, we described a potential pathway in which physical activity or fitness may influence dental caries through changes in SIgA. Exercise can both increase and decrease mucosal immunity depending on its intensity and context [20], and may also affect salivary gland function through autonomic regulation [21].

In the present study, physical activity was not positively associated with SIgA; instead, a small inverse association was observed. Although SIgA was positively associated with dentine caries, no indirect effect was detected in the mediation analyses. These findings do not support a mediating role of SIgA in the associations between physical activity or fitness and dentine caries. However, given the cross-sectional study design, the mediation analyses should be interpreted as exploratory rather than mechanistic.

Instead, the observed associations may reflect other mechanisms. Physical activity and fitness are often linked to healthier behaviors [5], such as better diet and oral hygiene, which may contribute to improved oral health outcomes. In addition, exercise may influence salivary secretion rate and buffering capacity [21], modulate stress-related hormones such as cortisol [22], and affect the oral microbial environment.

After adjustment, physical activity was not associated with dentine caries, whereas physical fitness retained a significant direct association with dentine caries. However, no significant indirect effects through SIgA were observed. This suggests that SIgA does not explain the observed association between physical fitness and dentine caries, and that other behavioral, socioeconomic, or biological mechanisms may contribute to the observed association.

In the adjusted models, SIgA was positively associated with DT, and in the descriptive analyses SIgA levels differed across DT and DMFT categories, despite its frequent characterization as protective at mucosal surfaces [14–17]. In negative binomial regression (Table 2), factors associated with restorative treatment need (DT) included lower tooth brushing frequency, suggesting poorer oral hygiene; lower household income, which may reflect limited access to health-related resources; tobacco use, which impairs oral immunity and healing [32]; and male gender, possibly due to behavioral or hormonal factors.

An alternative interpretation is that elevated SIgA reflects a reactive response to ongoing microbial challenge rather than a protective mechanism. Similar patterns have been described on other mucosal surfaces, such as the respiratory and gastrointestinal tracts, where SIgA secretion increases in response to microbial antigens [33]. A plausible explanation is that individuals with more caries also tend to have immunogenic bacteria in the caries biofilm. According to the ecological plaque hypothesis, repeated acidification of the dental biofilm leads to a dysbiotic microbial shift favoring aciduric and immunogenic bacteria, such as Streptococcus mutans, Lactobacillus spp., or other as-yet unidentified taxa, contributing to the cariogenic biofilm [34]. The proliferation of these species increases both the antigenic load and the exposure of oral mucosa to microbial components capable of stimulating mucosal immunity [6]. Therefore, individuals with more extensive caries could experience stronger activation of the secretory immune response, reflected in elevated SIgA levels.

Another potential pathway is that a dysbiotic biofilm associated with active dentine caries may expose the host to increased microbial challenge through microinvasion into dentinal tubules or low-grade systemic translocation of bacterial components, although these mechanisms are not as well established. It is also possible that other mediating factors play a role. Nonetheless, carious lesions could contribute to systemic immune activation, as suggested by recent reviews linking dental caries to broader systemic effects [35].

The positive association between SIgA and DT observed in this study contrasts with earlier research, which has typically reported a negative association between SIgA levels and DT [17], although some studies have also found positive or null associations [36]. In a recent systematic review and meta-analysis [17], lower SIgA levels were reported mainly in children and Asian populations, while evidence in adults was unclear due to the limited number of studies. Moreover, most of the included studies had methodological limitations such as small sample sizes, case–control designs, lack of adjustment for confounders, and inconsistent protocols for saliva collection and SIgA measurement.

In this study, systemic immunoglobulins were not significantly associated with dentine caries. Serum IgA showed only a marginal inverse trend (*p* = 0.082) (Table 2). These results suggest that dentine caries may be more closely linked to local mucosal immunity than to systemic immunoglobulin levels in this dataset, which is consistent with the notion that caries have a localized nature, in contrast to the systemic inflammation typically observed in periodontal disease.

Salivary SIgA and serum immunoglobulins represent distinct immunological compartments. SIgA is produced locally by plasma cells in mucosal tissues, whereas serum immunoglobulins reflect systemic immune activity. We did not assess correlations between salivary and serum IgA in this study; therefore, the relationship between these compartments warrants further investigation.

Strengths of this study include a large, population-based sample of Finnish middle-aged adults, calibrated dental assessments validated with a gold standard, and objective measurements of physical activity and fitness. Physical activity was assessed using a validated Polar Active accelerometer [37, 38], while physical fitness was estimated using heart rate recovery following a standardized step test. The inclusion of both salivary and serum immune markers adds valuable insight into the distinct roles of local and systemic immunity.

Limitations include the cross-sectional design, which precludes causal inference. Reverse causation is possible; for example, poor oral health could reduce physical activity due to pain or impaired quality of life. In addition, participation in the clinical examination was voluntary, and participants may therefore represent a subgroup with more favourable health behaviors, which could introduce selection bias. Focusing exclusively on 46-year-olds limits generalizability, as immune function, behavior, and caries risk may vary across the lifespan. Additionally, total immunoglobulin levels are crude markers that lack pathogen specificity, and caries, as a chronic outcome, may not align temporally with short-term fluctuations in immune or behavioral markers. It should also be noted that DMFT reflects cumulative lifetime caries experience rather than current disease activity, which may partly explain differences in associations observed for DT and DMFT. The number of participants varied slightly across statistical models, and we acknowledge that the reported consumption of beverages, candy, or snacks may not fully capture overall dietary behavior, but may instead reflect habitual sugar exposure rather than exact intake. Although BMI was included as a covariate, its relationship with dental caries was beyond the scope of the present study. In addition, we lack information on the general health status of the participants at the time of serum and saliva sampling, such as the presence of infections, which could potentially influence immunoglobulin levels. Saliva sampling conditions (e.g., timing and hydration) were not fully available, and the use of stimulated saliva may have influenced SIgA concentrations due to dilution effects. Furthermore, stimulated and unstimulated saliva differ in glandular origin and composition, and unstimulated saliva plays an important role in continuous mucosal and tooth surface protection. Therefore, our findings may not be directly comparable to studies using unstimulated saliva.

## Conclusions

In this dataset, SIgA did not mediate the associations between physical activity or physical fitness and dentine caries. While no association between physical activity and dentine caries was observed after adjustment, physical fitness retained a significant direct association with dentine caries, independent of SIgA. Notably, higher SIgA levels were associated with greater dentine caries in all models, suggesting that SIgA could serve as a marker of immune activation in response to caries rather than being a protective factor against caries, arguing against a uniformly protective interpretation of total SIgA. Serum immunoglobulins were not significantly associated with caries, suggesting that local immune responses may be more closely linked to dentine caries than systemic immunoglobulin levels in this dataset. Further research is needed to clarify the precise role of SIgA in caries progression.

## Supplementary Information

Supplementary Material 1.

## Data Availability

NFBC data are available from the University of Oulu, Infrastructure for Population Studies. Permission to use the data can be applied for research purposes via an electronic material request portal. In accordance with the EU General Data Protection Regulation (679/2016) and the Finnish Data Protection Act, the individual-level data cannot be openly shared, and access is granted only to qualified researchers upon application.
