# Supplementary material for: Salivary IgA and dentine caries in relation to physical activity and fitness: A cohort-based cross-sectional study
Source: Clin Oral Investig. 2026 Jul 15;30(8):326. doi: 10.1007/s00784-026-07035-y (PMC13369708; doi:10.1007/s00784-026-07035-y)
Supplement: Supplementary file 1 — Supplementary Material 1. [file 784_2026_7035_MOESM1_ESM.docx]

Supplementary Appendix

Title: **Salivary IgA and dentine caries in relation to physical activity and fitness: a cohort-based cross-sectional study**

Authors: Eero Blomster^1^, Marja-Liisa Laitala^1,2^, Sohvi Hörkkö^3^, Ramin Akhi^3^*, Tarja Tanner^1,2^*

Email: [Eero.blomster@oulu.fi](mailto:Eero.blomster@oulu.fi)

Clinical oral investigations

**Supplementary table 1**. One-way ANOVA of Salivary IgA and Serum IgA, IgG, and IgM (log-transformed for normality) across groups of tooth decay (ICDAS>3), caries status (DMFT), physical activity, and physical fitness, with post-hoc Tukey’s HSD tests.

| *Variables* | *p-value* | | *Tukey’s HSD group differences (p-value)* | | |
| --- | --- | --- | --- | --- | --- |
| *Salivary SIgA* |  | *1^st^ vs 2^nd^* | | *1^st^ vs 3^rd^* | *2^nd^ vs 3^rd^* |
| *DT* | 0,007 | 0,908 | | 0,005 | 0,015 |
| *DMFT* | <0,001 | 0,023 | | <0,001 | 0,006 |
| *Physical activity* | 0,031 | 0,028 | | 0,077 | 0,944 |
| *Physical fitness* | 0,601 | 0,599 | | 0,692 | 0,984 |
| *Serum IgA* |  | *1^st^ vs 2^nd^* | | *1^st^ vs 3^rd^* | *2^nd^ vs 3^rd^* |
| *DT* | 0,692 | 0,850 | | 0,848 | 0,694 |
| *DMFT* | 0,322 | 0,481 | | 0,307 | 0,795 |
| *Physical activity* | 0,042 | 0,175 | | 0,032 | 0,261 |
| *Physical fitness* | 0,050 | 0,042 | | 0,169 | 0,991 |
| *Serum IgG* |  | *1^st^ vs 2^nd^* | | *1^st^ vs 3^rd^* | *2^nd^ vs 3^rd^* |
| *DT* | 0,965 | 0,990 | | 0,978 | 0,962 |
| *DMFT* | 0,164 | 0,221 | | 0,191 | 0,881 |
| *Physical activity* | 0,123 | 0,931 | | 0,419 | 0,102 |
| *Physical fitness* | 0,405 | 0,404 | | 0,515 | 0,968 |
| *Serum IgM* |  | *1^st^ vs 2^nd^* | | *1^st^ vs 3^rd^* | *2^nd^ vs 3^rd^* |
| *DT* | 0,394 | 0,762 | | 0,523 | 0,790 |
| *DMFT* | 0,472 | 0,714 | | 0,439 | 0,755 |
| *Physical activity* | 0,097 | 0,954 | | 0,174 | 0,091 |
| *Physical fitness* | 0,474 | 0,909 | | 0,474 | 0,521 |
|  |  |  |  |  |  |

DMFT (Decayed, Missing, and Filled Teeth) was categorized to DMFT <11 (lowest), 11-19 (middle), and >19 (highest), and DT (Decayed teeth) to 0 (lowest), 1-3 (middle), and >3 (highest) dentine caries lesions. Physical activity and Physical fitness were categorized as follows: lowest 10% (1st group), middle 80% (2nd group), and highest 10% (3rd group).

**Supplementary fig.1**: Distribution of serum IgA, IgG, and IgM levels by three categories of DMFT, Tooth decay (ICDAS>3), Physical activity, and Physical fitness


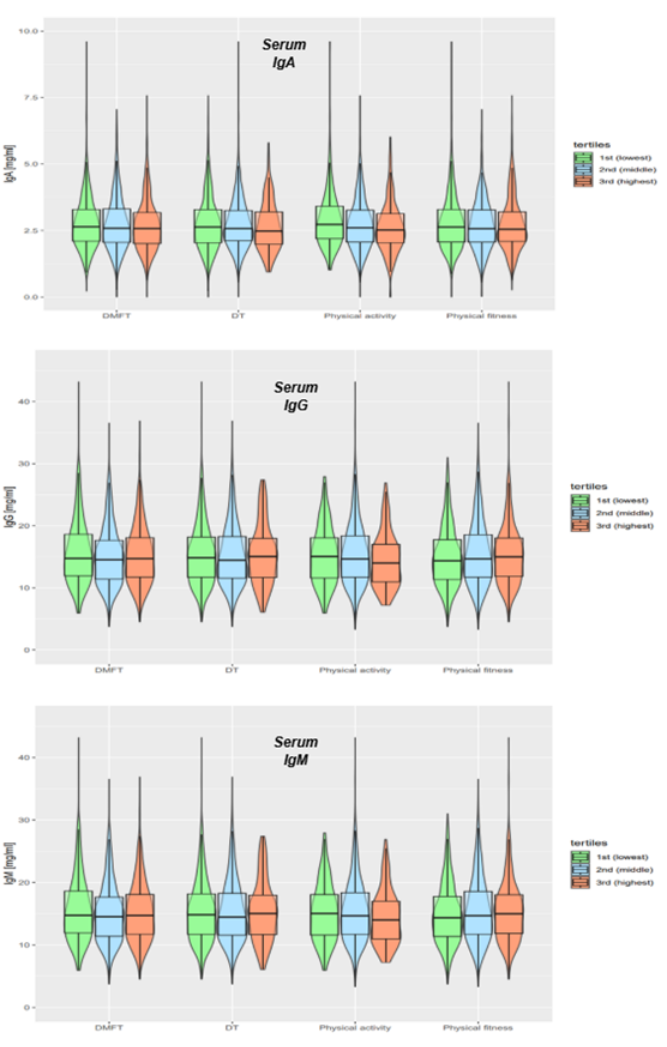


DMFT (Decayed, Missing, and Filled Teeth) was categorized to DMFT <11 (lowest), 11-19 (middle), and >19 (highest), and DT (Decayed teeth) to 0 (lowest), 1-3 (middle), and >3 (highest) dentine caries lesions. Physical activity and Physical fitness were categorized as follows: lowest 10% (1st group), middle 80% (2nd group), and highest 10% (3rd group).
